# The Ability of Probiotic Strain *Escherichia coli* O83:K24:H31 to Modulate Gut Homeostasis and Immune Function After Antibiotic-Induced Dysbiosis

**DOI:** 10.1007/s12602-025-10814-w

**Published:** 2025-11-19

**Authors:** Eliška Miková, Eliška Krčmářová, Viktor Černý, Lydie Sklenářová, Pavla Avramová, Eliška Protivová, Barbora Slunéčková, Muhammad Akhtar, Jan Věcek, Jan Procházka, Agnieszka Kubik-Zahorodna, Petra Petrásková, Olga Novotná, Helena Pelantová, Marek Kuzma, Irma Schabussova, Jiří Hrdý

**Affiliations:** 1 Institute of Clinical Immunology and Allergology, First Faculty of Medicine, Charles University and General University Hospital in Prague, Prague, Czech Republic; 2 Faculty of Science, Charles University, Prague, Czech Republic; 3 Institute of Specific Prophylaxis and Tropical Medicine, Centre for Pathophysiology, Infectiology and Immunology, Medical University of Vienna, Vienna, Austria; 4 Czech Centre for Phenogenomics, Institute of Molecular Genetics, Czech Academy of Sciences, 252 50 Vestec, Czech Republic; 5 Institute of Microbiology, Czech Academy of Science, Prague, Czech Republic

**Keywords:** Dysbiosis, Antibiotics, Probiotics, *E. coli*, Neutrophils

## Abstract

A healthy microbiome and a homeostatic interaction between the microbiome and the host immune system are essential for proper nutrition and overall health. Excessive use of antibiotics (ATB) can disrupt the healthy gut microenvironment, leading to dysbiosis, a condition linked to a wide range of disorders and diseases. Alterations in the composition and function of the microbiota have been associated with a broad spectrum of pathological conditions. In this work, we investigated the effect of ATB administration on microbiota composition and immune modulation, with a particular focus on neutrophil dynamics. We evaluated the capacity of the probiotic strain *Escherichia coli* O83:K24:H31 (EcO83) to mitigate ATB-induced dysbiosis and restore immune function. As expected, ATB treatment reduced microbiota diversity, which was partially restored by EcO83 supplementation. Furthermore, ATB administration affected the expression of tight junction proteins in the small intestine, an effect reversed by EcO83 treatment. Notably, our data indicate that ATB-induced dysbiosis accelerates neutrophil aging and reduces the release of neutrophils from the bone marrow. EcO83 supplementation counteracts these effects by promoting the influx of newly generated neutrophils into circulation. Overall, our findings confirm that ATB treatment disrupts gut microbiota homeostasis, adversely affecting immune function, including neutrophil turnover. However, probiotic supplementation with EcO83 can at least partially restore microbiome composition and immune homeostasis, highlighting its potential therapeutic application in mitigating ATB-induced dysbiosis.

**Supplementary Information:**

The online version contains supplementary material available at 10.1007/s12602-025-10814-w.

## Introduction

Excessive antibiotic (ATB) usage has a deleterious effect on microbiota [1, 2]. It is known that after ATB administration, a temporary dysbiosis occurs, possibly triggering immune system dysregulation [3, 4]. Upon steady state, commensal and beneficial microbes present in our physiological microbiota can prevent the overgrowth of pathobionts. But, during ATB treatments, these “good bugs” are also eliminated, and pathobionts can easily overgrow and bias immune responses towards proinflammatory, leading to the development of diseases (e.g., inflammatory bowel diseases) [5]. ATB intervention could negatively affect the long-term homeostatic interactions of the host's immune system with the microbiota; in the case of ATB-induced dysbiosis, chronic immunopathological responses may develop [6, 7]. Recently, early postnatal ATB administration has been shown to limit proper microbiota formation, which is linked with obesity development [8]. ATB administration has a profound effect on a broad spectrum of physiological functions, including immune system development and maintenance.

The mucosal microbiota profoundly shapes the development and polarization of the mammalian immune system [9]. Neutrophils, the most abundant population of leukocytes, present the first line of defense against pathogenic microorganisms. Upon encounter with a pathogen, neutrophils are immediately released from the bone marrow to fight against infectious agents. Neutrophils are professional phagocytes, which can efficiently eliminate ingested microbes with an abundance of enzymes (e.g., NADPH oxidase, myeloperoxidase, neutrophil elastase) and antimicrobial peptides (α-defensins 1–4) present in their granules.

Neutrophils, owing to their abundance and potent antimicrobial activity, are central to bacterial clearance in tissues and accumulate in large numbers in the inflamed intestine. Their infiltration into the lamina propria during intestinal inflammation is closely associated with Th17-mediated immune responses. Flannigan and his team [10] showed in an experimental mouse model that IL-17A drives, through the engagement of CXCR2, the expansion and migration of neutrophils to the ileum, where they control the numbers of segmented filamentous bacteria (SFB). They demonstrated that IL-23 is an important cytokine released after SFB colonization, which in turn induces the production of IL-22, which is linked to the secretion of antimicrobial peptides and, thus, the ability of neutrophils to control the numbers of bacteria in the intestine [10]. Besides that, IL-17A induces the production of granulocyte colony-stimulating factor (G-CSF), a critical granulopoiesis growth factor [11]. For a long time, it was thought that the enhanced presence of neutrophils was solely a hallmark of intestinal inflammation. Nevertheless, recent data show that neutrophils play a more complex role at the intestinal barrier and represent one of the important cell types maintaining mucosal homeostasis [12]. Components of intestinal bacteria (e.g., lipopolysaccharide, peptidoglycan) promote the maturation and priming of circulating neutrophils. On the contrary, some bacterial products (e.g., short-chain fatty acids) can inhibit neutrophil maturation. Neutrophils can, in turn, limit the number of bacteria by producing reactive oxygen species (ROS), antimicrobial peptides (AMP), and enzymes. Extensive ROS production can prevent bacterial translocation and dissemination throughout the body [13].

ATB treatment can affect neutrophil antimicrobial functions. In vitro experiments demonstrated that ATB administration can enhance antimicrobial killing by neutrophils [14]. Watanabe et al. showed that ATB administration can severely affect neutrophil trafficking to the gut and subsequently increase the degree of amebiasis [15]. They also showed that this phenomenon was caused by the decreased presence of CXCR2 on the neutrophil surface [15].

Importantly, it is established that neutrophils are a rather heterogeneous immune cell population with diverse functions ranging from pro-inflammatory to immunosuppressive, and that the disruption of proportions of individual neutrophil subsets can lead to chronic inflammatory diseases. Distinct subpopulations of neutrophils are characterized according to the cell surface expression of typical markers. These include CD11b, Ly6G, Ly6C, CXCR2, and CD62L in mice and CD11b, CD11c, CD15, CD16, CD62L, and CD66b in humans [16].

The beneficial effect of probiotics has been acknowledged, and the administration of probiotic strains is a rational way to promote the renewal of microbiota and mutual homeostatic interactions between the microbiota and the host immune system. Nevertheless, it is important to highlight that the beneficial effect of probiotics is highly strain-specific. Suitable strains used for the correction of ATB-disturbed microbial communities should thus be carefully selected. Notably, distinct probiotic strains have diverse capacities to modulate the immune system, ranging from pro-inflammatory and anti-infectious to immunoregulatory effects [17–19]. In addition to that, the study of the impact of probiotics on microbiota normalization in ATB-induced dysbiosis is challenging since we do not know the exact composition of normal/healthy microbiota. There is still ongoing scientific discussion about the definition of healthy gut microbiota, with limited evidence of the role of probiotics to renew ATB-disrupted microbiota [20]. Exempt from probiotics, fecal microbiota transplantation (FMT) has been proposed to be superior to restore microbiota composition upon ATB administration [21]. On the other hand, the necessity of any intervention to facilitate the microbiota renewal upon ATB administration is questionable since the microbiota itself has a quite dynamic nature (adaptation to dietary changes, circadian changes, changes during host ontogeny etc.) and can be restored spontaneously from resident microbiota present in crypts. Finally, discussion about the cause and consequence of distinct microbiota pattern associated with particular disease is still ongoing and further contributing to the complexity of microbiome research and possible ways of microbiome modulation.

Liu et al. showed that live probiotic bacteria *Lactobacillus johnsonii* can lower the severity of colitis and inhibit the formation of neutrophil extracellular traps (NETs) [22]. Deregulated enhanced formation of NETs can be pathological, for example, in autoimmune diseases, such as rheumatoid arthritis [23], diabetes mellitus [24], or systemic lupus erythematosus [25]. On the other hand, the probiotic *Escherichia coli* Nissle 1917 was a potent inducer of NETosis [26], suggesting that the inhibitory effect of probiotics on NET formation is strain-specific.

Taken altogether, neutrophils play a key role in intestinal inflammation as well as its resolution. As one of the cell types mediating the immune response to intestinal bacteria, they are important for the maintenance of healthy and balanced microbiota. We thus aimed to elucidate how ATB-induced dysbiosis affects immune function with a special focus on neutrophils and how the introduction of probiotics can normalize this. To understand the effect of ATB administration on the relationship between the microbiota and the immune system, we used a mouse experimental model, where dysbiosis was induced by ATB administration. We tested the capacity of the probiotic strain *Escherichia coli* O83:K24:H31 (EcO83) to correct the dysbiosis and renew mutual homeostatic interactions between microbiota and host immune system, preventing the development of undesirable chronic pro-inflammatory responses. Originally, EcO83 was administered to neonates to prevent nosocomial infections [27]. In addition to that, EcO83 has been shown to prevent allergy development [27] and to promote immunoregulatory responses, possibly limiting pro-allergic responses [28]. Findings show that EcO83 contributes to immune system maturation together with setting regulatory responses [29, 30]. Based on previous results, we evaluated the capacity of EcO83 to dampen inflammation by inducing regulatory responses and promoting gut barrier function in mice with ATB-induced dysbiosis.

## Materials and Methods

### Mice

BALB/cAnNCrl were bred in the Czech Center for Phenogenomics (Vestec, Czech Republic) or purchased from Velaz and kept in sterilized, filter-topped cages and fed autoclaved food with free access to clean water. Female mice aged between 8–11 weeks were used in the experiments. Mice were sacrificed by cervical dislocation. All mouse experiments were approved by the institutional review board of the First Faculty of Medicine, Charles University (IRB 1.LF-731) and the animal committee and executed according to good practice with animal models (MSMT-17298/2021–4).

### Antibiotic and Probiotic Treatment

Mice were divided into four groups: the control group (C), the *Escherichia coli* O83:K24:H31 (EcO83) group, the antibiotic (ATB) group, and the ATB + EcO83 group. Mice were treated with antibiotics introduced in sterile drinking water for two weeks. Control mice were given sterile water. The antibiotic mixture was added to the fresh drinking water and exchanged every 3 days. Ampicillin (1 g/L), neomycin sulphate (1 g/L), metronidazole (1 g/L), and vancomycin (0.5 g/L) (Sigma-Aldrich, USA) were diluted in sterile water to reach the final concentrations. The selection of ATB was based on several reports [31–33]. Following the antibiotic treatment, mice were treated with a probiotic bacterial strain, EcO83, every day for 5 consecutive days by intragastric gavage, at 5 × 10^8^ CFU in 200 μl of gavage buffer (PBS containing 200 mM NaHCO_3_ and 2% glucose), with a stainless-steel mouse gavage needle. EcO83 was cultivated as previously described [23]. Briefly, EcO83 was inoculated in Luria Bertani broth and cultivated for 6 h (37 °C upon agitation). After cultivation, bacterial cell culture was centrifuged, washed 2 times with PBS, and resuspended to a final concentration of 2.5 × 10^9^ CFU/ml in the gavage buffer.

### Blood, Bone Marrow, and Tissue Collection

Tissue samples were collected from euthanized mice. Blood was drawn from the lateral saphenous vein into a tube containing heparin (10 USP units/ml of blood) to prevent coagulation and centrifuged to collect plasma for analysis of cytokine concentration. Bone marrow was obtained from the femur by flushing with sterile non-supplemented cell culture medium (RPMI1640), gently dissociated by pipetting up and down, and filtered through a cell strainer (70 μm) to eliminate clumps and remaining tissue to obtain single cell suspension for further analysis. Intestinal sections (ileum and colon) devoid of stool and single-cell suspensions from mesenteric lymph nodes (mLN) were stored in RNAprotect Tissue Reagent (QIAGEN, USA) until RNA extraction.

### Microbiome Analysis from Stool Samples

Stool samples were collected directly from the distal part of the colon right after the mouse sacrifice. Samples were snap-frozen in liquid nitrogen and kept at −80 °C until further processing. Total DNA was extracted from the stool using the QIAmp Fast DNA Stool Mini Kit (Qiagen) according to the manufacturer's protocol. DNA quality and concentration were determined using NanoDrop. 16S rDNA metagenomic NGS libraries from the DNA samples and subsequent sequencing using the Illumina MiSeq v3 were performed at the Institute of Applied Biotechnologies a.s. Only samples that passed the Quality Control (A260/280: 1.6–2.1) were used to prepare the NGS libraries, which were sequenced on NovaSeq X Plus using the standard workflow. The microbial composition was analyzed using relative abundance, where the number of reads for each taxonomic level (phylum, class, order) was normalized to 100% of the total classified reads. Raw sequencing data are available in the Sequence Read Archive (SRA) under BioProject accession number PRJNA1226621 (cited 2025 Mar 10) available from: https://www.ncbi.nlm.nih.gov/sra/PRJNA1226621. Originally, 6–10 mice per group were included and presented in Figures unless an outlier value was excluded using Dixon´s test.

### Flow Cytometry Analysis

Neutrophil subsets were analysed in BM single-cell suspensions using flow cytometry. Cell suspensions were stained for the following cell surface markers: anti-mouse CD11b (clone M1/70, EXBIO, CZ), Ly6G (clone 1A8, BioLegend, USA), Ly6C (clone HK1.4, BioLegend, USA), CXCR2 (clone SA044G4, BioLegend, USA), CD62L (clone MEL-14, EXBIO, CZ), incubated for 10 min at room temperature in the dark, followed by red blood cell lysis and acquired immediately using BD FACS Canto II (Becton Dickinson). The representative gating strategy for the identification of neutrophil phenotype is shown in Supplementary Fig. 1.

### RNA Isolation, cDNA Library, and Quantitative Real-Time PCR

Total RNA was isolated from the mLN, ileum, and colon tissue using the RNeasy Mini Kit (QIAGEN, USA) according to the manufacturer’s instructions. The concentration and purity of isolated RNA was checked using Nanodrop (Thermo Fisher Scientific, USA) before following analyses. 0.5 μg of total RNA was reverse transcribed using a High-Capacity cDNA Reverse Transcription Kit (Thermo Fisher Scientific, USA) to create a cDNA library. Briefly, relative gene expression was quantified using a TaqMan gene expression assay (Applied Biosystems, USA) in tissue and cells. The list of TaqMan assays is provided in Table 1. Relative quantification of gene expression was related to the level of gene expression of beta-actin (*Actb*), used as a reference gene (housekeeping gene, endogenous control). The qPCR reactions were performed in duplicates using the LightCycler 480 Real-Time PCR System (Roche, Switzerland).

**Table 1:** List of probes used for analysis of genes of interest

Gene symbol	Assay ID	Gene name
*Actb*	Mm00607939_s1	Actin beta
*Cldn1*	Mm1342184_m1	Claudin-1
*Cldn5*	Mm00727012_s1	Claudin-5
*Il2*	Mm00434256_m1	Interleukin 2
*Il4*	Mm99999154_m1	Interleukin 4
*Il10*	Mm01288386_m1	Interleukin 10
*Il18*	Mm00434226_m1	Interleukin 18
*Ifng*	Mm01168133_g1	Interferon gamma
*Ocln*	Mm00500910_m1	Occludin
*Becn1*	Mm01265461_m1	Beclin-1
*Mki67*	Mm01278617_m1	Ki-67
*Map1lc3a*	Mm00458724_m1	Microtubule-associated protein 1 light chain 3 alpha

### Detection of Cytokines by ELISA

To confirm that changes observed in mRNA level are reflected in total cytokine milieu in sera, cytokines IL-1β, IL-8, and IL-18 were detected by ELISA using kits (IL-1β cat. no. 88–7261-88, ThermoFisher Scientific); IL-8 cat. no. MBS286946-96; IL-18 cat. no. MBS9135813-96, both purchased from MyBioSource) according to the manufacturer´s recommendations.

### Sample Preparation for SDS-PAGE Protein Electrophoresis and Western Blot

For western blot sample preparation, 15 mg of tissue preserved in RNAlater at −20 °C was used. The tissue was homogenized with an electric homogenizer in 300 μl of lysis buffer including protease inhibitors. The homogenized tissue was then incubated on ice for 20 min and centrifuged for 20 min at 4 °C and 20,000 g. The supernatant was transferred into a new microtube and stored at −20 °C. The prepared cell lysates were mixed with the required amount of 5 × Laemmli buffer, incubated in a heat block at 95 °C for 5 min, and then immediately used for protein SDS-PAGE electrophoresis.

For SDS-PAGE protein electrophoresis (Mini-PROTEAN® Tetra System, BioRad), a 12% separating (running) gel and a 5% stacking Tris–glycine gel were prepared. Samples were loaded at 8 μl per well, and the molecular weight marker at 3 μl per well. Electrophoretic separation was performed for 20 min at 80 V and 2 h at 140 V. Protein transfer from the gel onto a nitrocellulose membrane was carried out using the semi-dry blot method. The transfer was performed in a blotting cassette using the Trans-Blot Turbo Transfer System (BioRad) at 25 V, 1 A for 30 min.

Immunodetection of Proteins on the Membrane and Signal Visualization.

The nitrocellulose membrane with transferred proteins was washed of blotting buffer by rinsing in 0.1% (v/v) Tween-20 in PBS for 5 min on a shaker. The membrane was then blocked with 5% (w/v) non-fat dry milk in 0.1% (v/v) Tween-20 in PBS for 1 h with constant rocking. This was followed by incubation with the primary antibody diluted in blocking solution overnight at 4 °C (GAPDH, G9545, used at 1:2000 dilution; Claudin-1, SAB3500438, used at 1:500 dilution; Occludin, SAB3500301, used at 1:500 dilution; all Merck, Claudin-5, ABT45, used at 1:250 dilution, purchased from Millipore). The next day, the membranes were washed 4 times for 10 min each in 0.1% (v/v) Tween-20 in PBS on a shaker and then incubated with the secondary antibody diluted in blocking solution for 30 min at room temperature (secondary antibody, SAB3700972, used at 1:5000 dilution, Merck). Finally, the membranes were washed again 4 times for 10 min each in 0.1% (v/v) Tween-20 in PBS.

The resulting signal was evaluated by chemiluminescence using solution A (18 ml ddH₂O, 2 ml 1 M Tris–HCl (pH 8.5), 200 μl 250 mM luminol, 80 μl 90 mM p-coumaric acid (PCA)) + B (18 ml ddH₂O, 2 ml 1 M Tris–HCl (pH 8.5), 20 μl 30% H₂O₂), mixed in a 1:1 ratio in a 150 mm dish. The membrane was incubated in this solution for 30 s. The signal was visualized using the Amersham™ Imager 6000 (GE Healthcare Life Sciences). Signal quantification was performed using ImageJ Fiji software, and the intensity of bands was normalized to mixed sample of control mice.

### Detection of Short-Chain Fatty Acids

Aliquots (180 μl) of fecal extract, thawed at room temperature, were mixed with 20 μl of a 10% solution of trimethylsilyl propionic acid sodium salt (TSP) in D_2_O and transferred into 3 mm NMR tubes. All measurements were performed at 25 °C on a 600 MHz Bruker Avance III spectrometer equipped with a 5 mm cryoprobe. Short-chain fatty acids (SCFAs) were quantified using 1D projections of J-resolved experiments with presaturation, acquired with the following parameters: number of scans = 16, spectral width (SW) = 16 ppm, number of data points = 16 k, number of increments = 40, SW in the indirect dimension = 78.125 Hz, and relaxation delay = 2 s. Spectra were automatically phased, referenced to TSP, and normalized in the 10–0.5 ppm region (excluding the water resonance) using probabilistic quotient normalization (PQN) in NMRProcFlow software [34]. Relative quantification of acetate, propionate, and butyrate was based on their methyl resonances.

### Data Analysis and Statistics

Flow cytometry results were analyzed using FlowJo v10 software (TreeStar, USA) using appropriate single stain compensation and fluorescence minus one (FMO) controls to set a proper gating strategy. Gene expression of the target gene was quantified relative to the housekeeping gene *Actb* using the 2^−ΔΔCt^ method [35]. Data normality was assessed. One-way ANOVA was used for normally distributed data, while the Kruskal–Wallis test was applied for non-normal distributions. Outliers were identified using Dixon's test (p < 0.2). Analysis and graphical processing were performed using GraphPad Prism 8 (USA). Results are presented as mean ± SEM. Statistical significance: *p < 0.05; **p < 0.01; ***p < 0.001; ****p < 0.0001.

## Results

### Antibiotic Treatment Induces Changes In Microbiota Composition

**Fig. 1:**
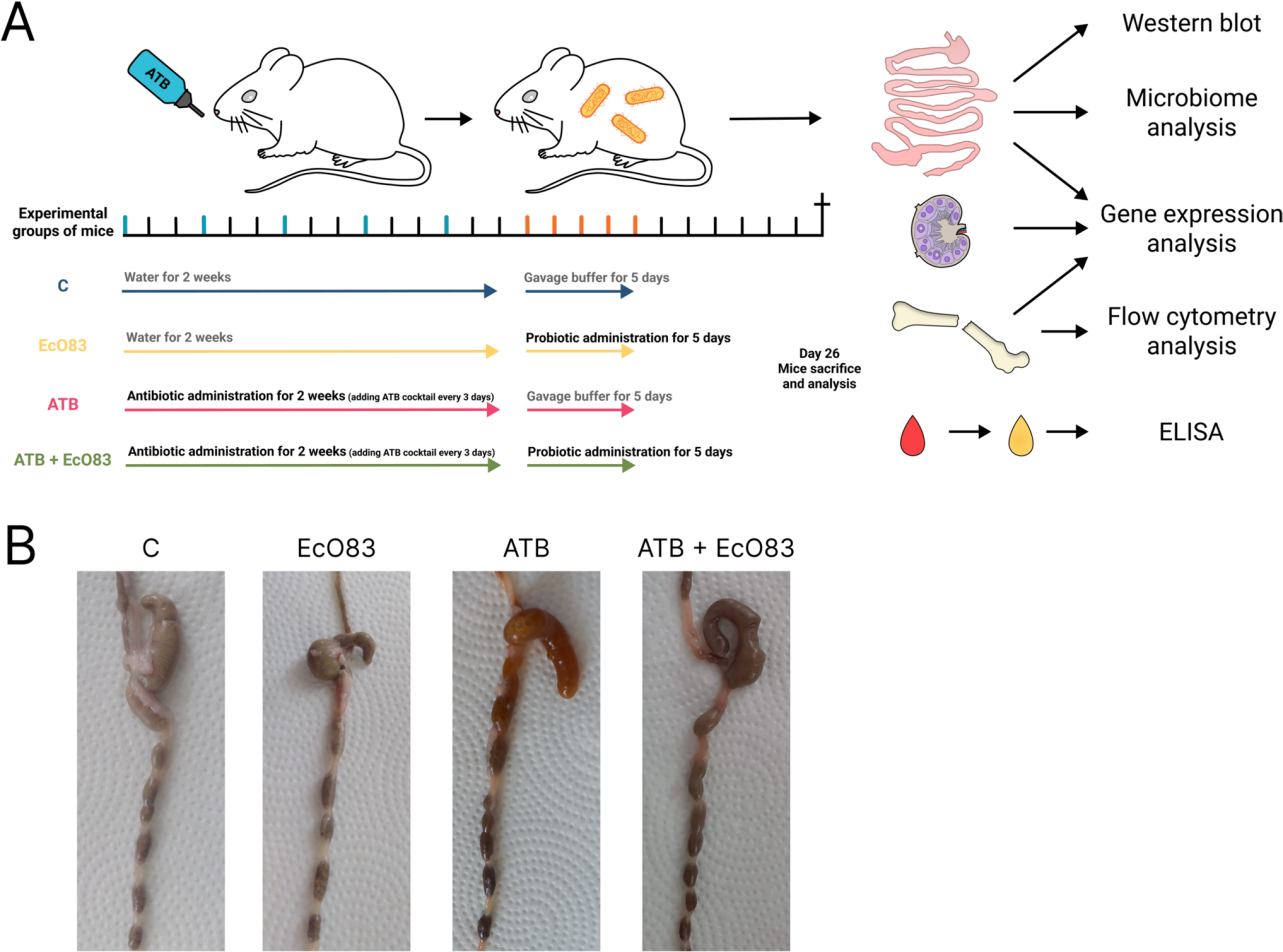
Experimental design and intestinal morphology. (**A**) Schematic representation of antibiotic-induced dysbiosis and subsequent probiotic treatment. BALB/c mice received a mixture of antibiotics (ampicillin 1 g/L, vancomycin 0.5 g/L, neomycin sulphate 1 g/L, and metronidazole 1 g/L) in drinking water for 2 weeks, with solution changes every 3 days, followed by oral gavage of probiotic EcO83 (5 × 10^8^ CFU in PBS containing 200 mM NaHCO_3_ and 2% glucose) for 5 consecutive days. Mice were sacrificed after the completion of probiotic treatment for sample collection. (**B**) Representative images showing the morphology of the caecum and adjacent small intestine (ileum) and part of the colon in the experimental groups of mice. Abbreviations: C (control), ATB (antibiotic mixture), ATB + EcO83 (antibiotic mixture + *Escherichia coli *O83:K24:H31), EcO83 (*Escherichia coli* O83:K24:H31)

To determine whether ATB treatment alters fecal microbiota composition and induces dysbiosis, adult mice were treated with a broad-spectrum antibiotic mixture (ampicillin, neomycin sulphate, vancomycin, and metronidazole) for one week (ATB group). Another group received EcO83 (ATB + EcO83 group) via oral gavage for five consecutive days following ATB treatment. Control groups consisted of untreated mice (C group) and mice supplemented with EcO83 only (EcO83 group), according to the experimental setup (Fig. 1A).

ATB treatment induced a drop in body weight, which was not statistically significant and normalized within one week regardless of EcO83 administration (data not shown). Although weight was restored, on the day of sacrifice, the caecum of ATB-treated mice appeared enlarged and dark (Fig. 1B) compared to the controls, a condition that was mitigated by EcO83 supplementation (Fig. 1B).

**Fig. 2:**
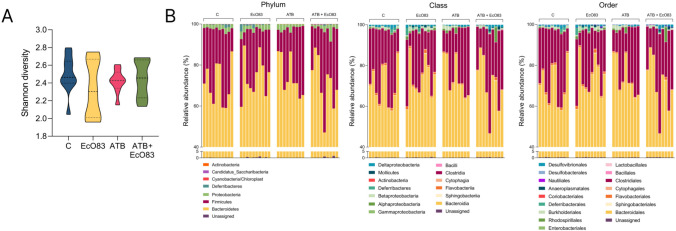
The bacterial composition on the day of sacrifice. (**A**) Alpha diversity of gut microbiota determined using the Shannon–Wiener index. (**B**) Stacked bar charts showing phylum, class, and order levels of bacterial composition. Abbreviations: C (control), ATB (antibiotic mixture), ATB + EcO83 (antibiotic mixture + *Escherichia coli* O83:K24:H31), EcO83 (*Escherichia coli* O83:K24:H31)

Microbiota composition was analyzed using 16S rDNA gene sequencing from stool samples. Both EcO83 and ATB treatment induce minor nonsignificant changes in the diversity and abundance of bacterial species as assessed by the Shannon–Wiener index (Fig. 2A). The fecal microbiome was classified at the phylum, class, and order levels (Fig. 2B). Marked dysbiosis was evident immediately after the end of ATB treatment (Supplementary Fig. 2). The significant impact of ATB and/or EcO83 administration on specific microbial phyla, classes, and genera is shown in Figure S3.

EcO83 supplementation significantly increased the abundance of *Candidatus* Saccharibacteria in both ATB-treated and non-treated mice (Supplementary Fig. 3 A**)**. The proportion of *Deferribacteres/Deferribacteriales/Deferribacteres* was reduced in ATB-treated mice, but EcO83 supplementation promoted their recovery (Supplementary Fig. 3). Conversely, *Flavobacteria* were significantly underrepresented in the gut microbiota of ATB-treated mice, and EcO83 had no effect on their restoration (Supplementary Fig. 3B). Similarly, *Sphingobacteria* levels were reduced following ATB treatment, with EcO83 supplementation having no impact on their restoration (Supplementary Fig. 3B, C). In contrast, EcO83 significantly promoted the growth of *Mollicutes*, *Anaeroplasmatales,* and *Nautiliales* as compared to control mice. Elevated levels of these taxa by EcO83 were also observed in ATB-treated mice (ATB + EcO83 group) (Supplementary Fig. 3B, C).

### EcO83 Administration Normalized Antibiotic-Induced Alterations in Gene Expression of Tight Junction Proteins in the Ileum

We aimed to evaluate the impact of dysbiosis on intestinal tissue damage and investigate whether EcO83 administration could promote recovery. To achieve this, we performed qPCR analysis on tissue samples from the ileum and colon, targeting genes involved in gut barrier function, including tight junction proteins Claudin-1 (*Cldn1*), Claudin-5 (*Cldn5*), and Occludin (*Ocln*), as well as the regulatory cytokine IL-10 (*Il10*).

**Fig. 3:**
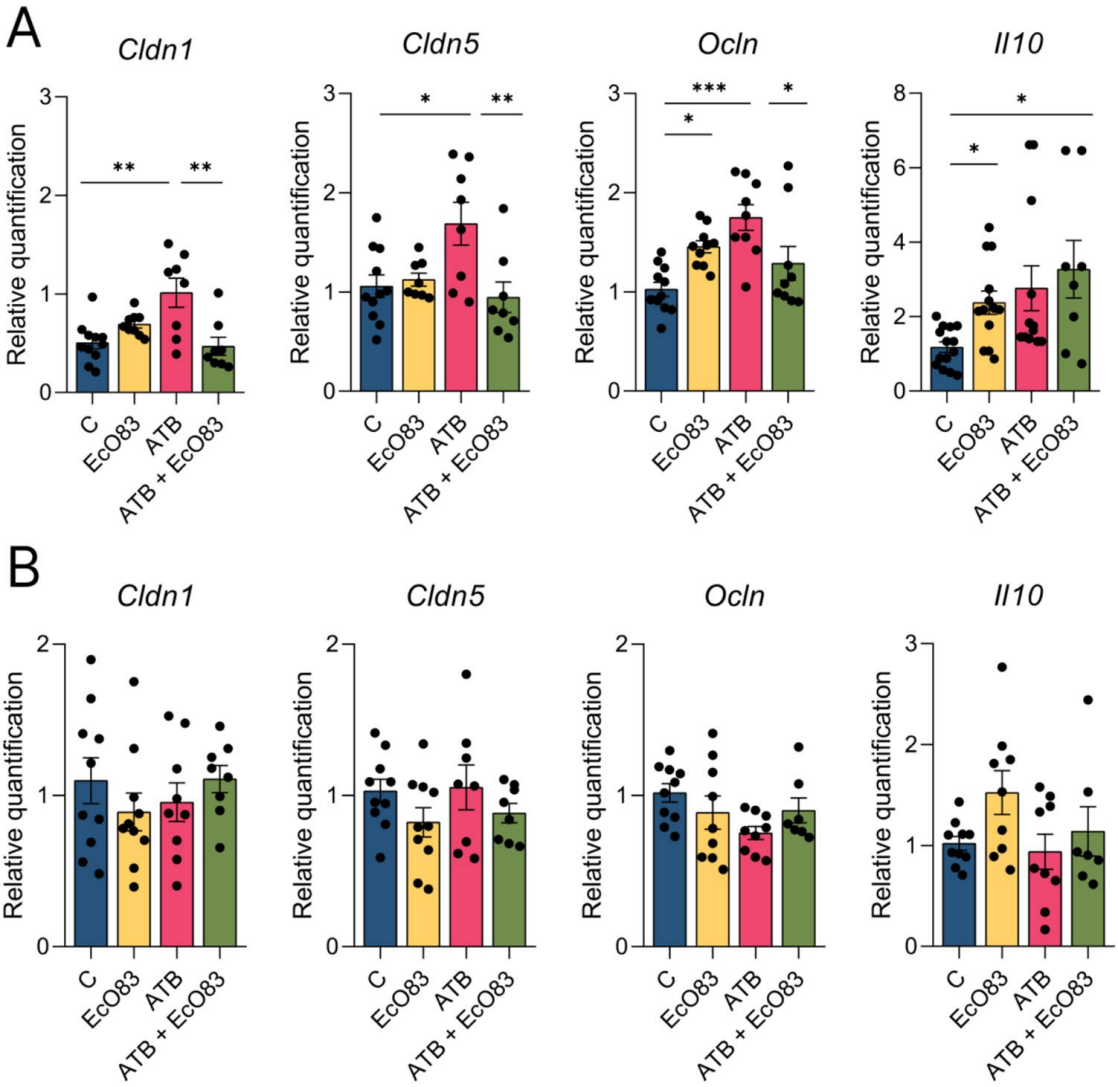
Gene expression analysis of markers associated with gut barrier function. Relative quantification of genes encoding tight junction proteins (*Cldn1*, *Cldn5*, *Ocln*) and the regulatory cytokine *Il10* was performed using qRT-PCR in the ileum (**A**) and colon (**B**) of mice treated with antibiotics and/or probiotic EcO83, compared to untreated controls. Data represent mean ± SEM. Statistical significance was determined using one-way ANOVA or Kruskal–Wallis test based on data distribution, where *p < 0.05, **p < 0.01, ***p < 0.001. Absence of significance indicators represents non-significant differences. Abbreviations: C (control), ATB (antibiotic mixture), ATB + EcO83 (antibiotic mixture + *Escherichia coli* O83:K24:H31), EcO83 (*Escherichia coli* O83:K24:H31).

Our analysis revealed significant modulation of gene expression in the small intestine of mice with ATB-induced dysbiosis (Fig. 3). The expression of tight junction genes was increased, particularly for *Cldn1* and *Cldn5* in ATB-treated mice, compared to control groups. EcO83 supplementation promoted the *Ocln* gene expression and enhanced the expression of the *Il10 gene*. Importantly, EcO83 supplementation also induced *Il10* expression in ATB-treated mice (Fig. 3A). Gene expression of tight junction proteins and *Il10* was also measured in the colon, but no significant changes were detected (Fig. 3B).

To confirm the impact of ATB and/or EcO83 on tight junction proteins at the protein level, the presence of Claudin-1, Claudin-5, and Occludin was determined in gut tissue samples. In the small intestine, we could detect changes corresponding to gene expression data, i.e., the highest levels of Claudin-1 and Claudin-5 were detected in the ATB-treated group, suggesting the compensatory effect of both elevated gene expression and protein translation for damaged gut barrier integrity. In contrast, Occludin levels in the small intestine did not differ among groups (Supplementary Fig. 4). Furthermore, no difference in the presence of tight junction proteins was detected in the colon on protein levels, data not shown.

### Cytokine Profile Remains Unchanged in Mesenteric Lymph Nodes After Antibiotic Treatment

**Fig. 4:**
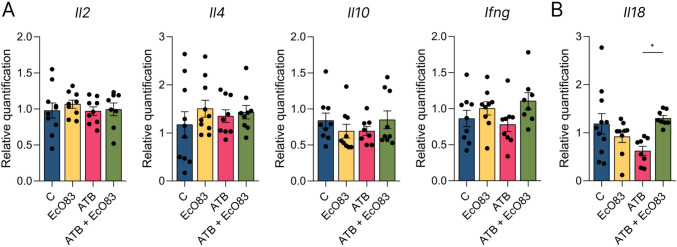
Gene expression of cytokines in mesenteric lymph nodes. Relative quantification of (**A**) *Il2*, *Il4*, *Il10*, and *Ifng*, and (**B**) *Il18* was performed using qRT-PCR in mice treated with antibiotics and/or probiotic EcO83, compared to untreated controls. Data represent mean ± SEM. Statistical significance was determined using one-way ANOVA or Kruskal–Wallis test based on data distribution, where *p < 0.05. Absence of significance indicators represents non-significant differences. Abbreviations: C (control), ATB (antibiotic mixture), ATB + EcO83 (antibiotic mixture + *Escherichia coli* O83:K24:H31), EcO83 (*Escherichia coli* O83:K24:H31)

Since we observed changes in the expression of ileal epithelial proteins, we investigated whether antibiotic treatment influenced cytokine expression in the mesenteric lymph nodes, which drain the gut epithelium. Interestingly, no significant changes were detected in any of the measured cytokines (Fig. 4), except for the normalization of IL-18 levels following EcO83 administration in ATB-treated mice (Fig. 4), a cytokine linked to neutrophil function and trafficking.

To assess potential systemic immune alterations, we also measured concentrations of inflammatory cytokines in serum. However, no significant changes in IL-1β, IL-8, or IL-18 serum levels were observed (Supplementary Fig. 5), indicating that antibiotic treatment did not induce a systemic pro-inflammatory response in our setting.

### EcO83 Administration Partially Corrected the Antibiotic-Induced Increased number of CD62L^−^CXCR2^−^ neutrophils

Finally, we sought to determine whether ATB treatment and EcO83 administration affected neutrophil phenotype. BM cell suspensions were analyzed for neutrophil surface markers, identifying neutrophils as CD11b^+^Ly6G^+^Ly6C^−^ granulocytes (gating strategy, Supplementary Fig. 1).

**Fig. 5:**
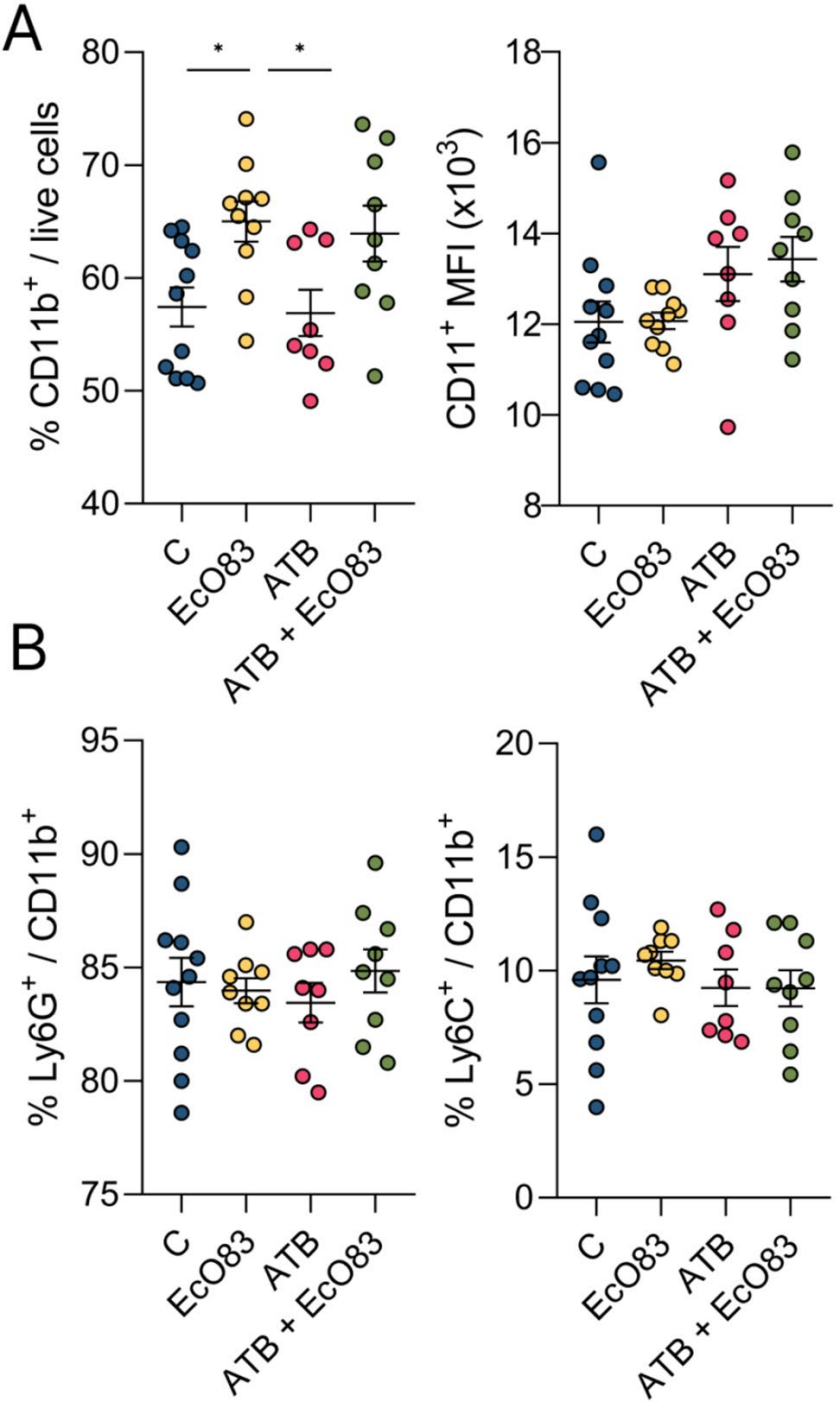
Analysis of neutrophil phenotype in bone marrow. Flow cytometric analysis of (**A**) CD11b^+^ cells percentage and their mean of fluorescence intensity (MFI), and (**B**) percentage of Ly6G^+^ granulocytes and Ly6C^+^ monocytes/macrophages within the CD11b^+^ population in mice treated with antibiotics and/or probiotic EcO83, compared to untreated controls. Data represent mean ± SEM. Statistical significance was determined using one-way ANOVA or Kruskal–Wallis test based on data distribution, where *p < 0.05. Absence of significance indicators represents non-significant differences. Abbreviations: C (control), ATB (antibiotic mixture), ATB + EcO83 (antibiotic mixture + *Escherichia coli* O83:K24:H31), EcO83 (*Escherichia coli* O83:K24:H31)

Although there was an increased number of CD11b^+^ cells in EcO83-treated mice, the overall mean fluorescence intensity (MFI) of CD11b remained comparable (Fig. 5A). Additionally, no significant changes were observed in the percentage of neutrophils (Ly6G^+^), which constituted the largest proportion of CD11b^+^ cells, or in the percentage of monocytes (Ly6C^+^) (Fig. 5B).

**Fig. 6:**
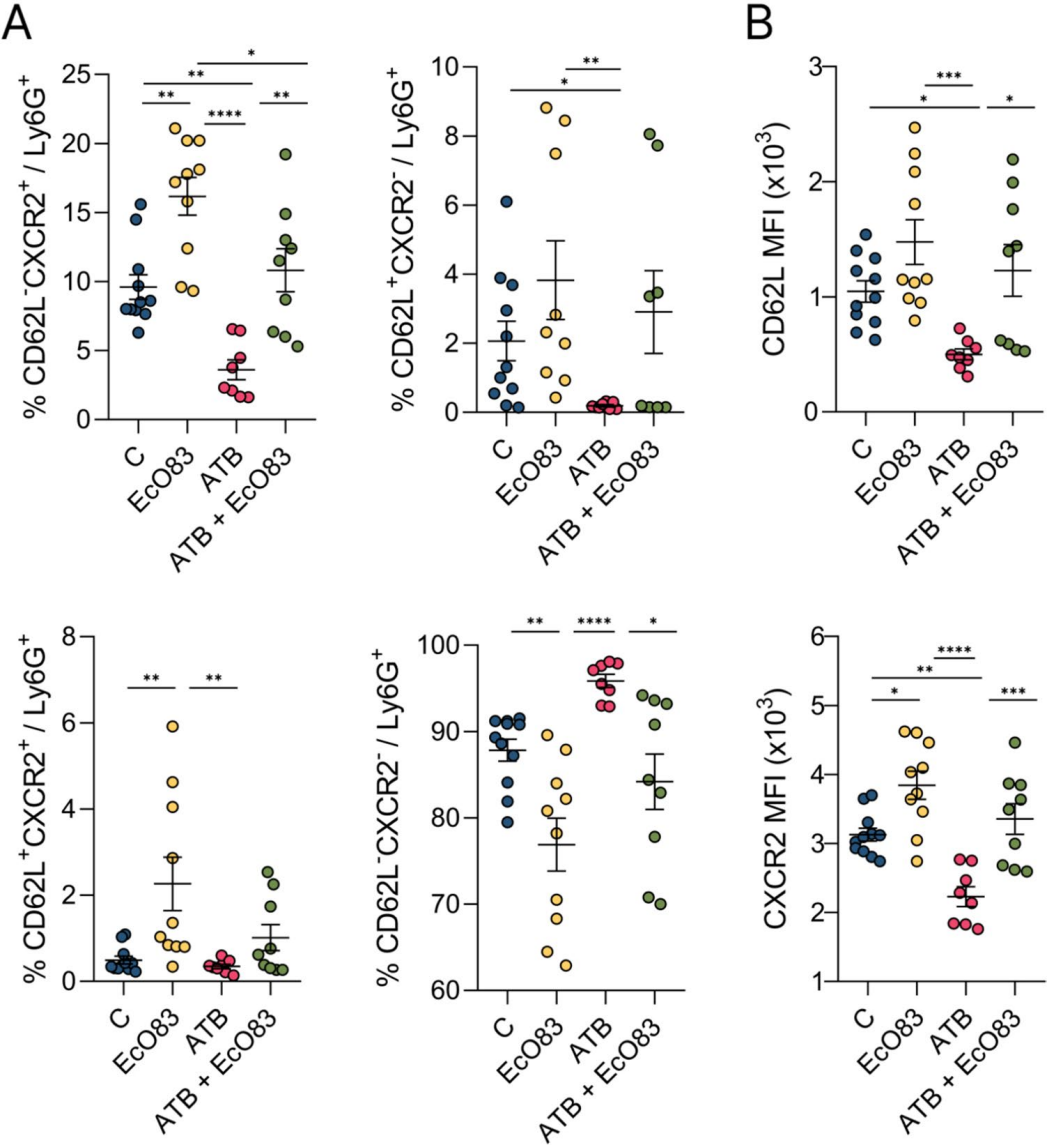
Analysis of neutrophil subpopulations in bone marrow. Flow cytometric analysis of (**A**) neutrophil subsets within the Ly6G^+^ population defined as CD62L^−^CXCR2^+^, CD62L^+^CXCR2^−^, CD62L^+^CXCR2^+^, and CD62L^−^CXCR2^−^, and (**B**) mean of fluorescence intensity (MFI) of CD62L and CXCR2 on Ly6G^+^ cells in mice treated with antibiotics and/or probiotic EcO83, compared to untreated controls. Data represent mean ± SEM. Statistical significance was determined using one-way ANOVA or Kruskal–Wallis test based on data distribution, where *p < 0.05, **p < 0.01, ***p < 0.001, ****p < 0.0001. Absence of significance indicators represents non-significant differences. Abbreviations: C (control), ATB (antibiotic mixture), ATB + EcO83 (antibiotic mixture + *Escherichia coli* O83:K24:H31), EcO83 (*Escherichia coli* O83:K24:H31)

The most profound differences were observed in the expression of the adhesion molecule CD62L and the IL-8 receptor CXCR2 on Ly6G^+^ neutrophils (Fig. 6). ATB treatment induced loss of CD62L expression, while EcO83 administration had the opposite effect. ATB-treated mice had a slightly increased number of CD62L^−^CXCR2^−^ double-negative cells, a change that was reversed to control levels upon probiotic administration (Fig. 6A, bottom right). When antibiotic treatment was followed by EcO83 intervention, the neutrophil phenotype was restored to its original state (Fig. 6B). To identify the possible impact of dysbiosis on proliferation or turnover rate, gene expression of markers associated with proliferation and autophagy was determined by qPCR. We were not able to detect a significant impact of ATB and EcO83 administration on changes in gene expression of *Mki67*, *Becn1,* and *Map1lc3a* (Supplementary Fig. 6).

### Short-chain fatty acid detection in feces

To confirm the capacity of EcO83 supplementation to modify SCFA production, SCFA were detected in feces at two time points. The first time point was immediately upon the last dose of EcO8, and there were only minor nonsignificant changes in the relative quantification of SCFA among groups (Supplementary Fig. 7 A). Determination of SCFA in feces at the second time point interval at the end of the whole experiment has not proved any impact of EcO83 nor ATB on SCFA production (Supplementary Fig. 7B).

## Discussion

The suitability of probiotic supplementation during ATB treatment remains an area of active debate. In this study, we investigated the ability of EcO83 to restore microbiota composition and immune function following ATB-induced dysbiosis in a murine model.

The prolonged dysbiosis triggered by ATB administration could lead to altered immune responses characterized by low-grade chronic inflammation supporting inflammatory bowel disease development. Therefore, the potential of probiotics to promote the restoration of microbiota composition and mutual homeostatic interactions between microbiota and the host immune system will be beneficial for the host's health. EcO83 supplementation has no significant impact on microbiota composition 12 days after ATB administration. No effect or even adverse effect of probiotic supplementation has been reported previously when probiotic supplementation prolonged diarrhoea and dysbiosis, documented by a lower number of microbial species detected in stool samples of patients [21, 36]. However, it is important to highlight that in these studies, a mixture of 11 bacterial species with a scarce probiotic effect documented by previous studies was used. In addition to that, according to the recommendation of ISAPP (International Scientific Association for Probiotics and Prebiotics), the probiotic strains should be reported unambiguously, i.e., including particular strain annotation. It is important to highlight that the probiotic effect is highly strain-specific and probiotic effect ranges from immunostimulatory to immunosuppressive/immunoregulatory [37]. Therefore, the selection of appropriate probiotic strains for the effects desired in a particular context (i.e., the correction of a concrete pathology) is critical. In our study, EcO83 has been selected due to its immunoregulatory capacity [28–30], which is desirable to suppress pro-inflammatory responses occurring during dysbiosis. Nevertheless, we could not demonstrate a clear effect of EcO83 supplementation on higher microbiota diversity (Fig. 2A). To confirm that our model of ATB administration is able to cause dysbiosis, microbiota composition has been analyzed immediately after ATB administration; Supplementary Fig. 2 clearly demonstrates a huge dysbiosis in both ATB and ATB + EcO83 groups before EcO83 administration. The absence of significant changes in alpha diversity (Shannon–Wiener index) following EcO83 supplementation could be partly due to the prolonged interval between the last dose of antibiotic administration and the day of sacrifice, and partly to functional redundancy within the microbiota as well as strain-dependent effects. In a healthy intestine, obligate anaerobes like *Firmicutes* and *Bacteroidetes* thrive in low-oxygen environments. Dysbiosis disrupts this delicate balance, leading to a decline in these anaerobes and a concurrent increase in facultative anaerobes such as *Enterobacteriaceae*, often associated with elevated ROS [38]. Immediately after antibiotic treatment, we observed a marginal rise in *Enterobacteriales*, a characteristic sign of dysbiosis—a state associated with reduced microbial diversity and increased *Proteobacteria*, particularly within the *Enterobacteriaceae* family. Although *Bacteroidetes* and *Firmicutes* levels decreased following antibiotic exposure, they independently returned to baseline values, demonstrating their remarkable resilience [39]. In our study, no delay of microbiota normalization has been observed in the EcO83-supplemented group. Moreover, no difference among groups (C, EcO83, ATB, ATB + EcO83) has been demonstrated, confirming that probiotic administration has no impact on the microbial community [40, 41].

Nevertheless, EcO83 increased the abundance of *Candidatus* Saccharibacteria, a finding of particular relevance for metabolomics, as this phylum has been identified as having the strongest impact on the metabolome associated with Alzheimer's disease [42]. Importantly, Saccharibacteria belong among potent producers of SCFA (acetate and lactate) [43, 44], highlighting the possible beneficial effect of EcO83 supplementation on the correction of ATB-induced dysbiosis. While EcO83 demonstrated the ability to support *Candidatus* Saccharibacteria colonization independently, the most pronounced effects were observed when mice received antibiotic treatment prior to probiotic supplementation. A comparable pattern emerged for the orders *Coriobacteriales* and *Nautiliales*, suggesting a potential synergistic interaction between antibiotic pretreatment and probiotic administration. EcO83 can elevate *Deferribacteres*, which belong to the phylum the most impacted by ATB administration in our study. Deferribacteres are known for their capacity to form various metabolites, including SCFA (e.g., acetate, lactate) [45, 46]. Association of an increased proportion of *Deferribacteres* in piglets with improved antioxidant function was documented in a study supplementing mothers with probiotics. The potential of probiotics to promote *Deferribacteres* and limit inflammation has been demonstrated previously [47, 48]. On the other hand, Panpetch et al. demonstrated an increased presence of *Deferribacteres* in a mouse model of dysbiosis triggered by dextran sulphate solution together with *Candida* and *Klebsiella pneumoniae* administration [49] and IL-1β expression in the gut in a mouse model of Diet-Induced Obese Mice [50]. Decreased levels of *Flavobacteria* were shown to be associated with IBD [51], but EcO83 supplementation was not able to restore diminished *Flavobacteria* after ATB treatment.

In our study, only a single strain of probiotic EcO83 has been used, and possibly a mixture of carefully selected probiotic strains can be more beneficial and/or impact a complex microbial community in mice without ATB treatment. This observation agrees with a previous study of early postnatal EcO83 supplementation, where no change in the microbiota of ten-year-old children has been reported [30]. However, even complex mixtures of probiotics have only a marginal effect on microbiota composition in healthy volunteers, as reviewed by Kristensen [40]. On the other hand, several reports highlight the impact of probiotics on microbial communities [52, 53]. This discrepancy can be explained by various probiotic strains used, dose (CFU) of probiotics, single strain versus complex mixture of probiotic strains, duration of probiotic supplementation, and dietary, ethnic, and socioeconomic differences of probands among the studies.

On the other hand, the beneficial effect of probiotics is not merely mediated by changes in microbiota composition measured by changes in alpha diversity, richness, or evenness. The beneficial effect of probiotics can be mediated by the production of SCFA directly by probiotic strains administered or the capacity of probiotic strains to trigger SCFA production [54–58]. In our study, the presence of SCFAs was assessed in fecal samples at two time points: at the cessation of EcO83 administration and on the day of mouse sacrifice. A nonsignificant trend toward elevated SCFA levels was observed in EcO83-treated mice; however, no difference was detected at the time of sacrifice. Including additional time points might be beneficial to better capture the period reflecting the most prominent changes in SCFA production following EcO83 supplementation. The other beneficial effect of probiotics involves the promotion of tight junction protein expression, leading to an increase in gut barrier function [59, 60]. We have demonstrated the capacity of EcO83 to promote tight junction protein expression in neonates supplemented by EcO83 [29]. Previous studies have shown that ATB administration decreases the expression of various genes, tight junction-associated proteins, namely Claudin-1, Occludin, and Zonulin-1 in the intestine [61] and Claudin 3,4 in the colon [62]. Subsequent administration of probiotics normalized the expression of Zonulin-1 and Occludin [63]**.** Previous research from our laboratory showed that early postnatal colonization of EcO83 increased the expression of the tight junction genes *Cldn* and *Ocln* as well as *Il10*, thereby supporting intestinal barrier function [29]. In the current study, the capacity of EcO83 to promote tight junction protein expression and restore gut barrier function in mice treated with ATB has been tested as well. In contrast to our hypothesis, the most elevated gene expression of all tight junction proteins has been detected in the ileum of mice treated with ATB, suggesting the increased gene expression is a compensatory effect for disturbed gut barrier function in an effort to renew the functionality of the gut barrier. EcO83 was able to promote gene expression of tight junction proteins, documenting the capacity of probiotics to promote gut barrier function [64, 65]. Our hypothesis that the most prominent expression of tight junction proteins is a compensatory effect of ongoing inflammation is further supported by decreased gene expression of tight junction proteins in the ATB + EcO83 group. Expression of tight junction proteins on the mRNA level was confirmed on the protein level using Western blot analyses. The most predominant presence of Claudin-1 and Claudin-5 was detected in the small intestine of ATB-treated mice. Nevertheless, it will be important to perform functional studies to determine whether these elevated gene expression and protein presence are sufficient for renewal of gut barrier function e.g., by FITC dextran permeability test [66].

IL-10 represents a cytokine with immunoregulatory function, preventing the development of pro-inflammatory responses. EcO83 was able to promote *Il10* expression, which is in line with our previous observations [28–30, 67]. Importantly, mice treated with ATB and EcO83 exerted increased gene expression of *Il10* as well*,* documenting the potential of EcO83 to limit inflammation triggered by ATB-induced dysbiosis. Surprisingly, neither ATB nor EcO83 modified the gene expression of tight junction proteins in the colon (Fig. 3B).

The capacity of probiotics to modulate adaptive immune responses has been described previously [68, 69]. Therefore, we investigated the impact of ATB and/or EcO83 administration on the local immune system in MLN. To our surprise, no tremendous changes in gene expression of selected cytokines have been documented. Only IL-18 was lowered in the treated group, and EcO83 supplementation normalized the expression of IL-18 to the level of control mice, highlighting the role of EcO83 in triggering anti-infectious immune responses [70, 71]. On the other hand, IL-18 can contribute to inflammatory responses [72]. Finally, IL-18 also plays an important role in gut microbiota monitoring and homeostasis, and its dysregulation could lead to microbiota dysbiosis and accelerate disease progression. Furthermore, the dysbiosis observed in *Il18*^−/−^ mice contributed to their increased susceptibility to *Listeria* infection [73]. However, our data of IL-18 concentration in sera are not supporting the link between IL-18 and microbiota changes in our experimental model. Therefore, additional experiments are needed to confirm the role of IL-18 in shaping microbiota composition and/or the impact of dysbiosis on triggering IL-18. This highlights the importance of fine-tuning immune responses with emphasis on appropriate regulatory mechanisms.

The other goal of the study was to characterize the impact of ATB-induced dysbiosis on the proportional and functional characteristics of neutrophils as cells of the first line of defense. Since the majority of neutrophils is located in the bone marrow, we investigated the proportion of particular neutrophil subsets there by flow cytometry. Both probiotic-treated groups (EcO83 alone and ATB + EcO83) possessed elevated levels of CD11b^+^ in bone marrow. ATB-treated mice have a significantly increased proportion of CD62L^−^ neutrophils associated with the typical phenotype of old senescent and exhausted cells. EcO83 supplementation was able to normalize the levels of CD62L^−^ neutrophils to the values found in healthy control mice. Altered granulopoiesis triggered by ATB can be demonstrated by the level of CXCR2, which was decreased in ATB-treated mice compared to control healthy mice. Diminished levels of CXCR2 in ATB-treated mice have already been published [15], and our study confirmed the potential effect of ATB on lowering the migration capacity of neutrophils to the site of infection/inflammation. Importantly, the effect of the probiotic strain *Bacillus polyfermenticus* in lowering the severity of colitis and promoting neoangiogenesis was dependent on CXCR2 and IL-8 signalling [74]. Together, these results suggest that lower CD11b, along with decreased CD62L and CXCR2, indicate a reduced capacity for rolling, adhering to endothelial tissues, and migrating into target tissues, which might result in impaired local immune responses. Antibiotic treatment contributes to this altered neutrophil profile, potentially compromising the immune system's ability to effectively respond to infections. Interestingly, EcO83 was able to restore granulopoiesis in ATB-treated mice. These results highlight the capacity of EcO83 to normalize granulopoiesis. We have tried to identify different proliferation rates and/or turnover in neutrophils, but we were not able to find a distinct pattern of gene expression of selected markers (*Mki67*, *Becn1*, *Map1lc3a*) in neutrophils from distinct groups. Additional studies are needed to better analyse the impact of ATB-induced dysbiosis on various parameters associated with granulopoiesis. The limitation of the current study is short duration of ATB administration, which should be extended in future follow-up studies.

## Conclusion

While EcO83 did not significantly modify microbiota diversity, it played a critical role in restoring gut barrier integrity and immune balance. The probiotic counteracted ATB-induced changes in neutrophil subsets, promoted *Il10* expression, and normalized *Il18* expression levels. These findings emphasize the importance of strain-specific probiotic effects beyond microbiota composition. Further research is needed to elucidate the mechanisms by which probiotics modulate immune responses and their potential applications in ATB-associated dysbiosis, with special focus on cytokine changes reflecting microbiota modulation.

## Supplementary Information

Below is the link to the electronic supplementary material.

Supplementary file1 (DOCX 1482 KB)

## Data Availability

Raw sequencing data are available in the Sequence Read Archive (SRA) under BioProject accession number PRJNA1226621 (cited 2025 Mar 10) available from: https://www.ncbi.nlm.nih.gov/sra/PRJNA1226621.
