# Supplementary material for: The Ability of Probiotic Strain Escherichia coli O83:K24:H31 to Modulate Gut Homeostasis and Immune Function After Antibiotic-Induced Dysbiosis
Source: Probiotics Antimicrob Proteins. 2025 Nov 19;18(4):5673–87. doi: 10.1007/s12602-025-10814-w (PMC13341875; doi:10.1007/s12602-025-10814-w)
Supplement: Supplementary file 1 — Supplementary file1 (DOCX 1482 KB) [file 12602_2025_10814_MOESM1_ESM.docx]

**Supplementary Figure 1: Flow cytometry gating strategy for analysis of bone marrow neutrophils.** Sequential gating shows the initial cell population based on forward and side scatter (FSC-A vs. SSC-A), followed by singlet identification (FSC-A vs. FSC-H) and subsequent analysis of neutrophil subsets based on cell surface marker presence (CD11b, Ly6C, Ly6G, CXCR2, CD62L). The top row demonstrates the gating strategy for stained samples, while the bottom row shows the corresponding FMO (Fluorescence Minus One). Representative plots from one sample are shown.

**
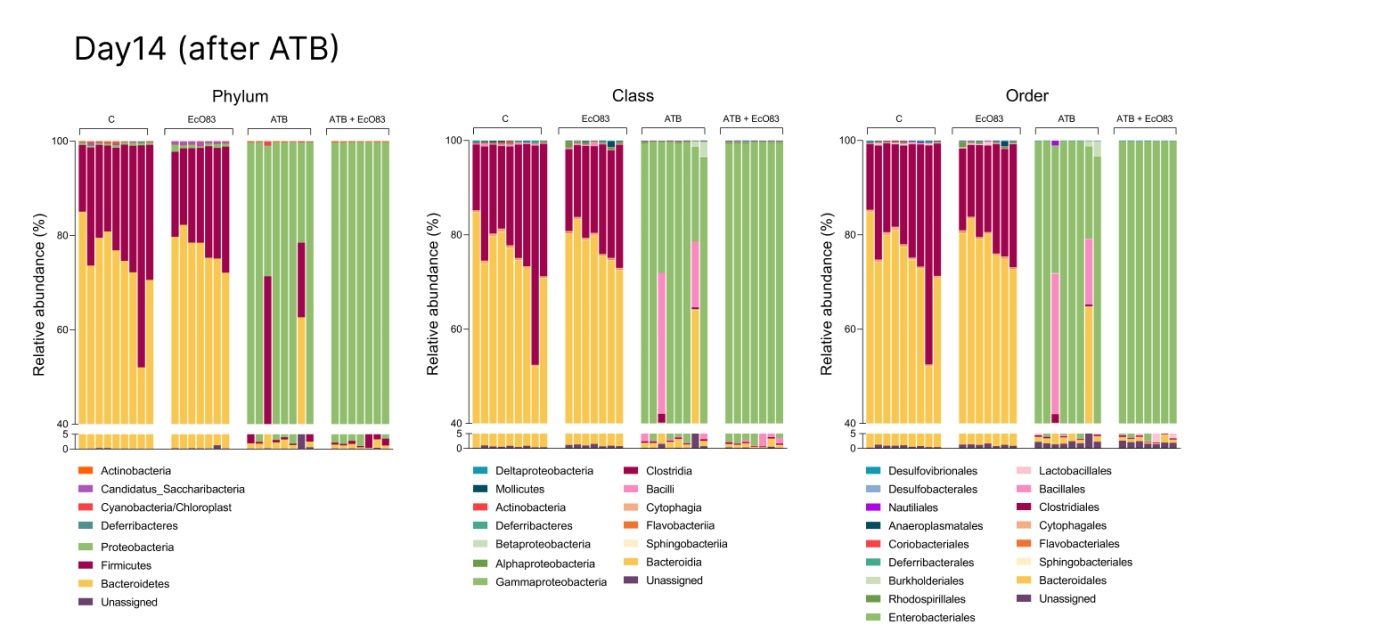
**

**Supplementary Figure 2: Analysis of bacterial composition at day 14 (i.e., immediately after antibiotic treatment).** Stacked bar charts showing relative abundance at phylum, class, and order levels in all experimental groups of mice. Data represent the mean relative abundance of bacterial taxa. Abbreviations: C (control), ATB (antibiotic mixture), ATB + EcO83 (antibiotic mixture + *Escherichia coli* O83:K24:H31), EcO83 (*Escherichia coli* O83:K24:H31).

**
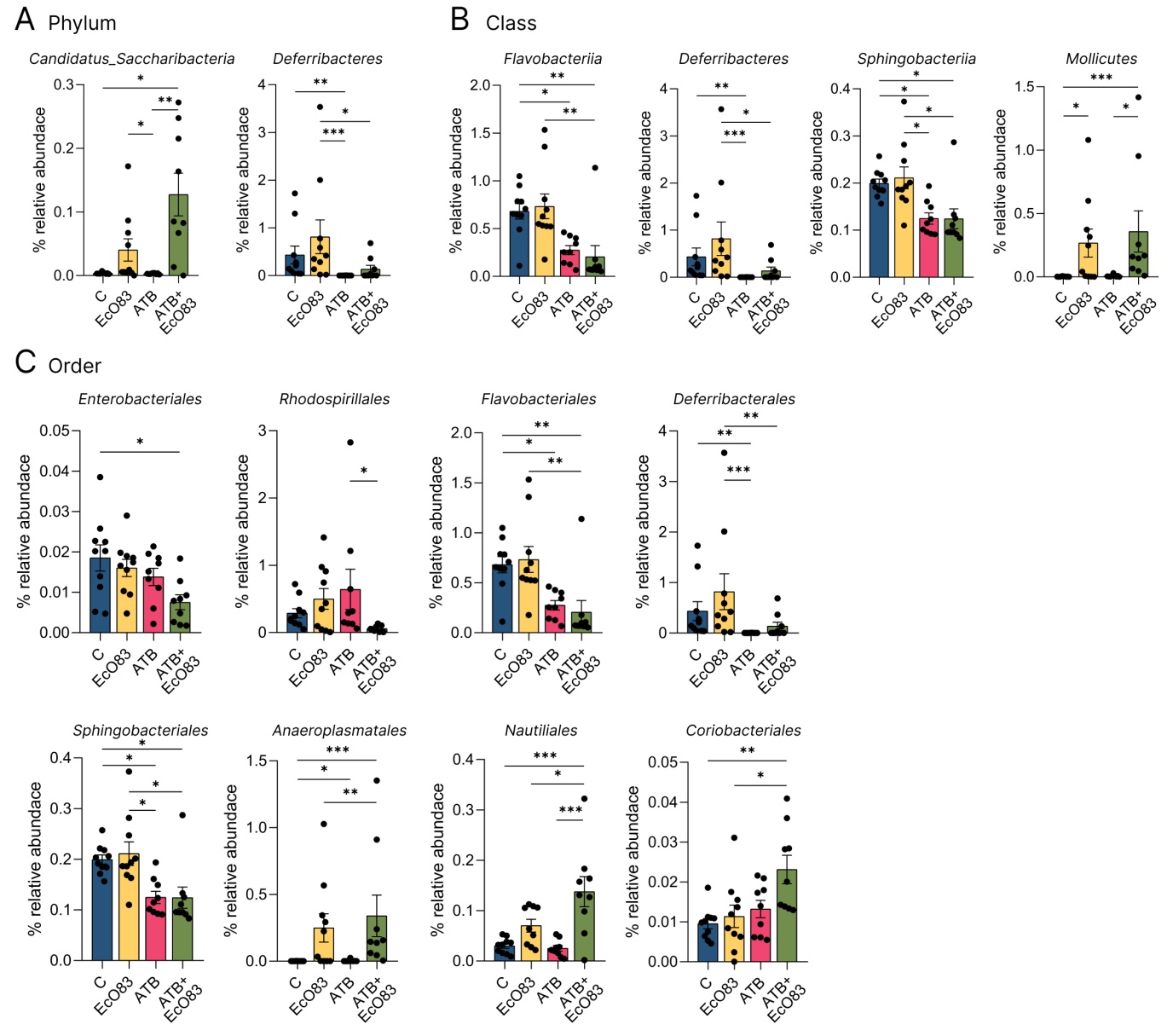
**

**Supplementary Figure 3: Relative abundance of significantly altered bacterial taxa on the day of sacrifice.** **(A)** Changes in the relative abundance of bacterial phyla *Candidatus_*Saccharibacteria and *Deferribacteres*. **(B)** Differences in bacterial classes *Flavobacteriia*, *Deferribacteres*, *Sphingobacteriia*, and *Mollicutes*. **(C)** Alterations in bacterial orders *Enterobacteriales*, *Rhodospirillales*, *Flavobacteriales*, *Deferribacterales*, *Sphingobacteriales*, *Anaeroplasmatales*, *Nautiliales*, and *Coriobacteriales* in all experimental groups of mice. Data are presented as mean ± SEM. Statistical significance was determined using one-way ANOVA or Kruskal-Wallis test based on data distribution, where *p<0.05, **p<0.01, ***p<0.001. Absence of significance indicators represents non-significant differences. Abbreviations: C (control), ATB (antibiotic mixture), ATB + EcO83 (antibiotic mixture + *Escherichia coli* O83:K24:H31), EcO83 (*Escherichia coli* O83:K24:H31).


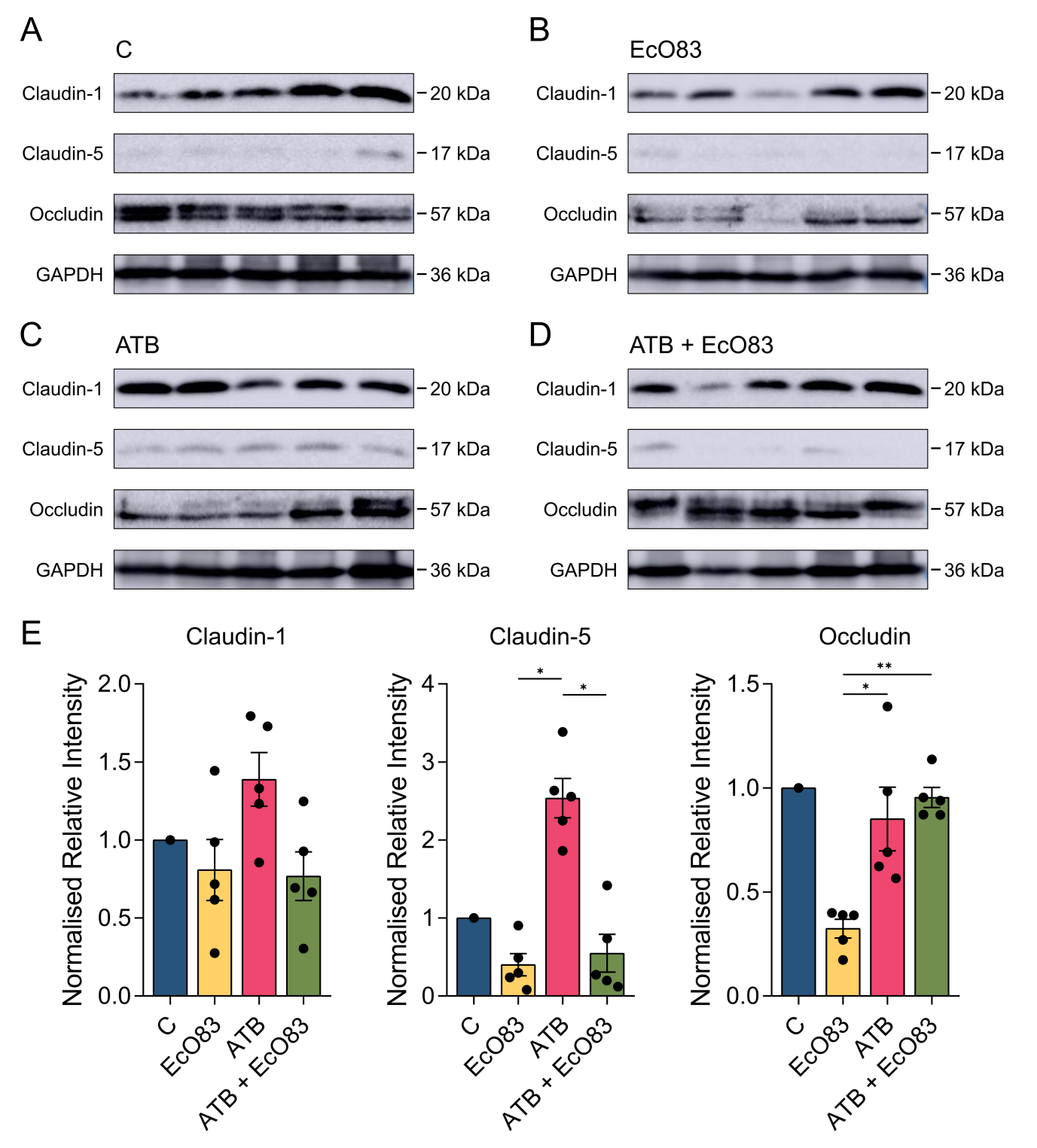


**Supplementary Figure 4: Detection of tight junction proteins using Western Blot.** **(A-D)** The levels of tight junction proteins (Claudin-1, Claudin-5, Occludin) of all experimental groups of mice were quantified in the small intestine using Western blot. **(E)** Semiquantitative analyses of target proteins relative to GAPDH and normalized to the controls are shown. Abbreviations: C (control), ATB (antibiotic mixture), ATB + EcO83 (antibiotic mixture + *Escherichia coli* O83:K24:H31), EcO83 (*Escherichia coli* O83:K24:H31).


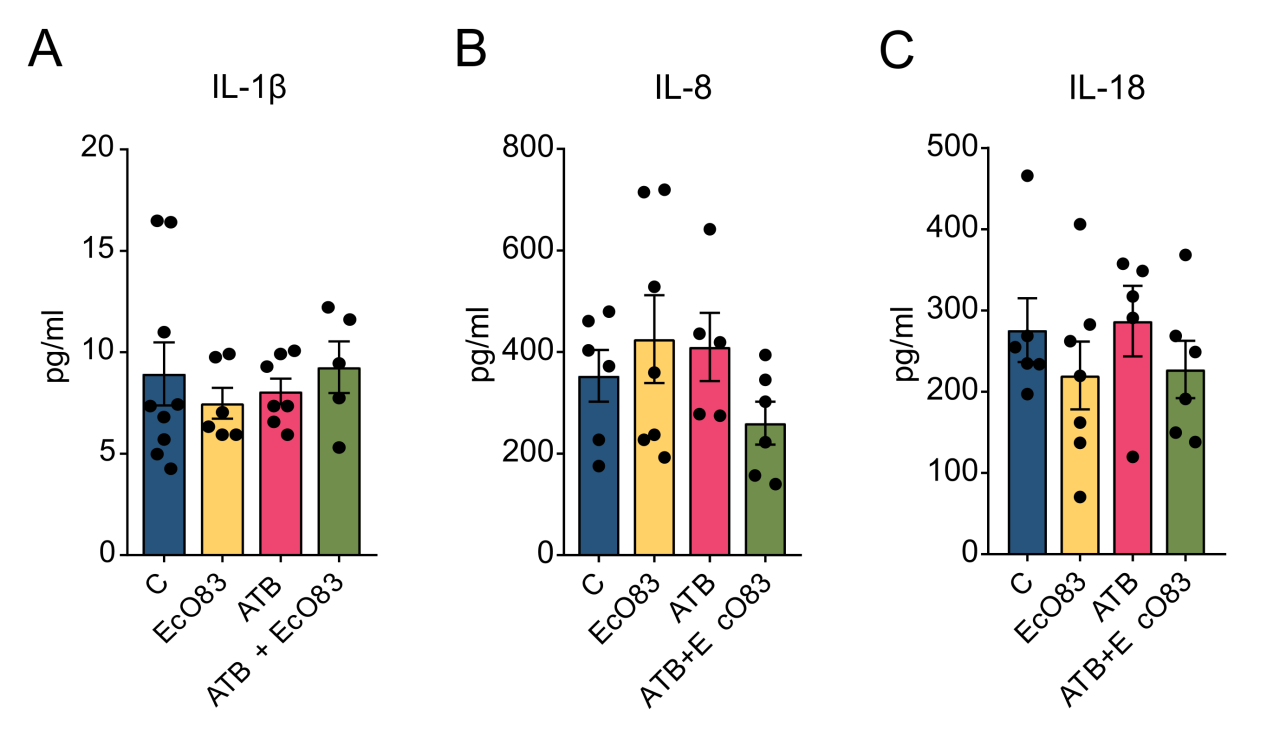


**Supplementary Figure 5: Detection of cytokine concentration in sera.** Concentration of **(A)** IL-1β **(B)** IL-8**,** and **(C)** IL-18 was measured by ELISA in the experimental groups of mice. Data represent mean ± SEM. No significant differences were observed among groups. Abbreviations: IL (interleukin), C (control), ATB (antibiotic mixture), ATB + EcO83 (antibiotic mixture + *Escherichia coli* O83:K24:H31), EcO83 (*Escherichia coli* O83:K24:H31).


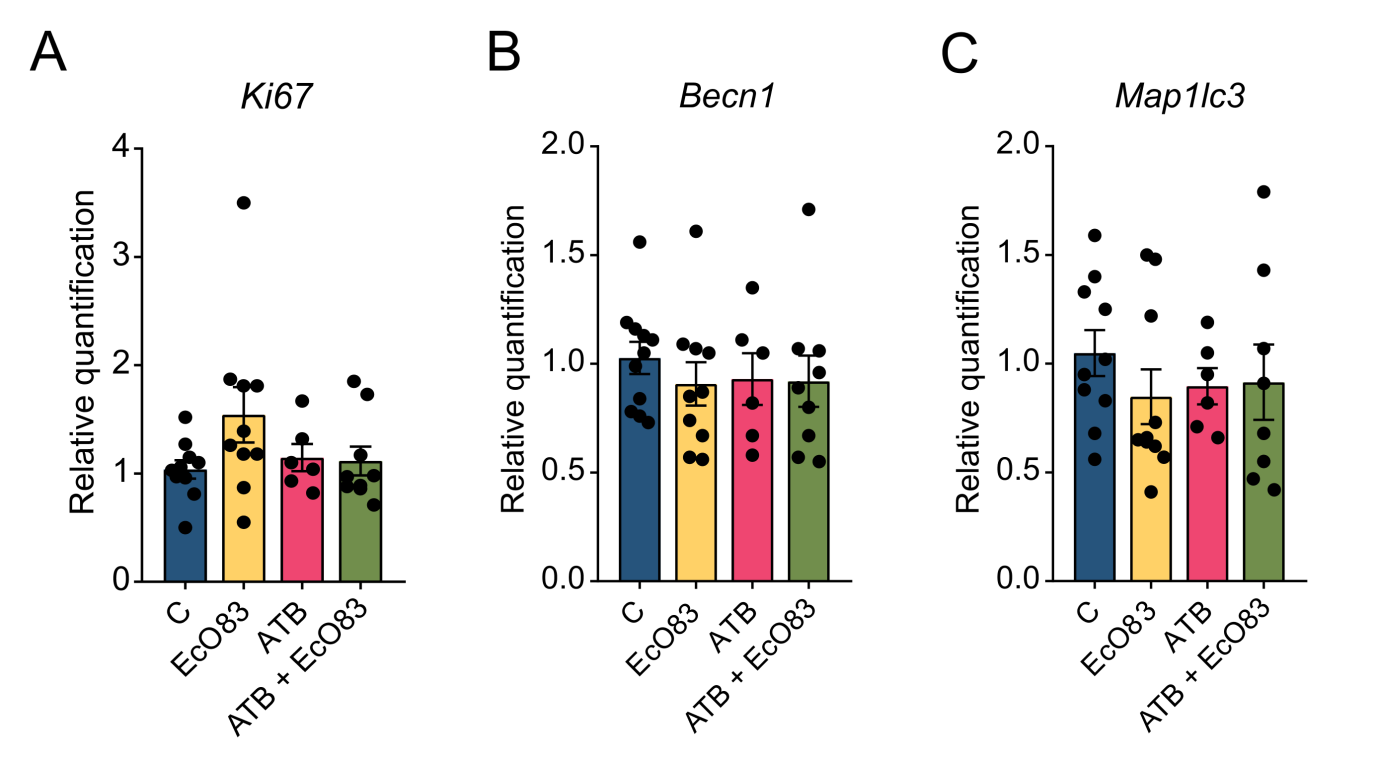


**Supplementary Figure 6: Evaluation of the impact of dysbiosis and probiotic supplementation on cell proliferation and turnover rate using quantitative real-time PCR.** Relative gene expression of **(A)** proliferation marker *Mki67*, and turnover markers **(B)** *Becn1*, and **(C)** *Map1lc3a*. Abbreviations: C (control), ATB (antibiotic mixture), ATB + EcO83 (antibiotic mixture + *Escherichia coli* O83:K24:H31), EcO83 (*Escherichia coli* O83:K24:H31).


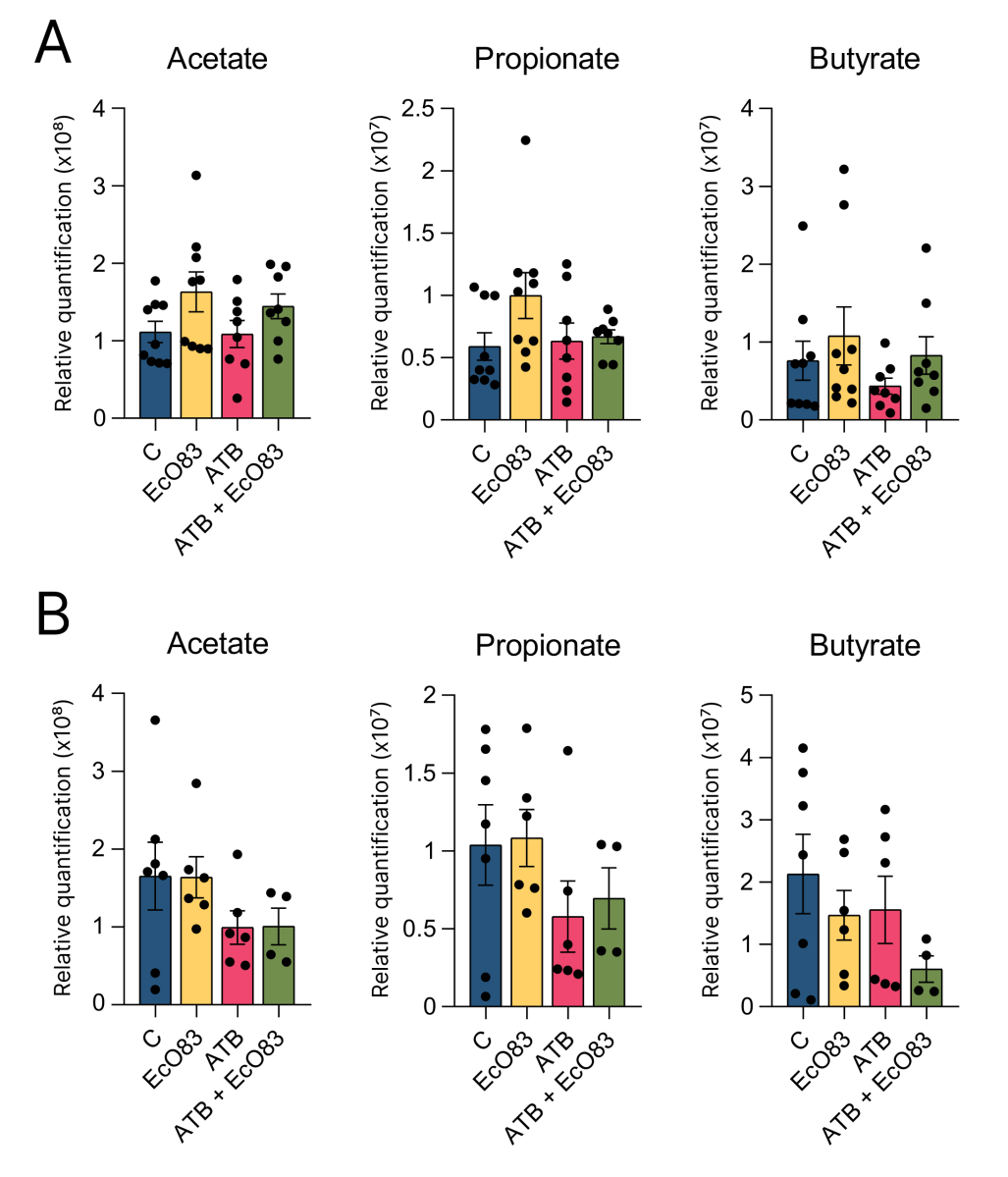


**Supplementary Figure 7: Detection of short-chain fatty acids in feces using NMR.** Relative quantification of acetate, propionate, and butyrate presence after the period of EcO83 administration (Day 19, **A**) and on the day of experiment termination (Day 26, **B**) in all experimental groups of mice. Abbreviations: C (control), ATB (antibiotic mixture), ATB + EcO83 (antibiotic mixture + *Escherichia coli* O83:K24:H31), EcO83 (*Escherichia coli* O83:K24:H31).
